# Nitrogen and Sulfur Co‐Doped Graphene‐Like Carbon from Industrial Dye Wastewater for Use as a High‐Performance Supercapacitor Electrode

**DOI:** 10.1002/gch2.201900043

**Published:** 2019-10-02

**Authors:** Yannan Lin, Hui Chen, Yulin Shi, Gang Wang, Long Chen, Fu Wang, Shiqi Li, Feng Yu, Lili Zhang

**Affiliations:** ^1^ Key Laboratory for Green Processing of Chemical Engineering of Xinjiang Bingtuan School of Chemistry and Chemical Engineering Shihezi University Shihezi 832003 P. R. China; ^2^ School of Environmental Science and Engineering Shanghai Jiao Tong University Shanghai 200240 P. R. China; ^3^ Xinjiang Shenbang Environmental Engineering Co., Ltd. Shihezi 832003 P. R. China; ^4^ Institute of Chemical and Engineering Sciences Agency for Science Technology and Research Jurong Island Singapore 627833 Singapore

**Keywords:** co‐doping, industry dyes, supercapacitors, wastewater

## Abstract

Nitrogen and sulfur co‐doped graphene‐like carbon (N,S‐GLC) is successfully prepared in a one‐step hydrothermal reaction of glucose with industrial dye wastewater followed by chemical activation. The nitrogen and sulfur are sourced entirely from the industrial wastewater. The process not only provides an alternative way of treating industry wastewater, but also offers a green route for recovering energy from the waste in the form of chemicals. The resultant N,S‐GLC shows a good degree of graphitization, a high specific surface area (1734 m^2^ g^−1^), and moderate heteroatom doping (N: 2.1 at%, S: 0.7 at%). The N,S‐GLC electrode displays high specific capacitance of 275 F g^−1^ at a current density of 0.5 A g^−1^ with a retention of 65.4% at 20 A g^−1^ in 6 m KOH. Moreover, the assembled symmetrical supercapacitor cell shows a capacitance of 38 F g^−1^ at a current density of 0.5 A g^−1^, which is equivalent to an energy density of 6.4 Wh kg^−1^ at a power density of 275.0 W kg^−1^. This approach provides an alternative and sustainable way of fabricating heteroatom‐doped graphene‐like carbon materials for use in high‐performance supercapacitors.

## 1 Introduction

Due to increased energy demands and intensified environmental concerns worldwide, there is an urgent need to develop eco‐friendly, low cost, and high‐performance energy storage devices.^1^, ^2^, ^3^ The supercapacitor is considered to be a promising energy storage device because of its fast charge and discharge, long cycle stability, and high power‐density compared with other storage devices.^4^, ^5^ Supercapacitors can be divided into pseudocapacitors and electrical double‐layer capacitors (EDLCs) based on their different charge storage mechanisms.^6^ EDLCs depend on the reversible adsorption and desorption of electrode ions at the interface between the electrode and the electrolyte. Until now, tremendous effort has been devoted to improving the capacity of supercapacitors by developing new kinds of electrode materials, including carbon‐based materials.^7,8^

Carbon compounds, such as graphene,^9^, ^10^ carbon nanotubes,^11^ and porous carbons,^12^, ^13^, ^14^ have received extensive attention as electrode materials in EDLCs. Given the need for the development of cost‐effective, well‐performing electrode materials,^15^ carbon materials that have a high specific surface area and hierarchical porous structures are an efficient way of obtaining satisfactory electrochemical performance when used as the electrodes in EDLCs.^16^, ^17^ Additionally, heteroatom doping with nitrogen (N) and sulfur (S) can enhance the capacitance by promoting the wettability and pseudocapacitive properties of the specialized carbon materials.^18^, ^19^ At present, the preparation of N and S co‐doped porous carbon is achieved by using N and S containing compounds. Recently, Li et al. reported fabricating a porous carbon using a potassium hydroxide (KOH)‐activated willow‐catkin process followed by N‐ and S‐doping using thiourea. The doped carbon had a specific capacitance of 249 F g^−1^ at 0.5 A g^−1^ in a 6 m KOH electrolyte.^20^ Kong et al. reported on N and S co‐doped graphene using ammonium thiocyanate (NH_4_SCN), which delivered a capacitance of 209 F g^−1^ at 10 A g^−1^ in a 6 m KOH electrolyte.^21^ Despite the beneficial increase in capacitance this type of doping produces, the current method for the preparation process is generally complex, time‐consuming, and results in low yields, which has largely limited its use to industrial applications. In addition, synthetic chemicals have been the main source of N and S dopants in previous methods, which increases the resource input required for manufacturing doped carbon materials. Therefore, the production of high‐performance, functional, porous carbon using green and efficient methods using renewable and sustainable feedstocks is a highly desirable future direction for the manufacturing of supercapacitors.^22^, ^23^, ^24^ Our approach has several advantages: 1) this method provides an alternative way of treating industry wastewater when producing useful doped carbon materials; 2) it offers a green route of recovering energy from the waste; 3) no additional N and S containing chemicals are required to produce doped porous carbon in our approach. With N and S dopants, new electrochemically active sites are generated. The Faraday reaction of the surface N, S functional group can increase the specific capacitance of the porous carbon material.

In recent years, an increasing demand for synthetic dyes has resulted in large amounts of wastewater generated by the textile and dyeing industries. This wastewater is composed mainly of aromatic heterocyclic compounds, which are difficult to degrade and harmful to human health,^25^, ^26^ meaning it is necessary to pretreat the wastewater before discharging to the environment. Adsorption is the most widely used method for treating this type of wastewater.^27^ In previous reports, research has focused on using various kinds of activated carbon as the adsorbents,^28^, ^29^ while little effort has been devoted to recovering energy from wastewater. The organic compounds that are rich in N and S present in the wastewater could support a new route of treatment that yields energy while preparing heteroatom‐doped graphene‐like carbon with the heteroatoms from the dye wastewater.

Herein, we present a new approach to generate graphene‐like porous carbon that is co‐doped with N and S via a hydrothermal reaction and subsequent chemical activation of the glucose in the dye wastewater, and its potential application to energy storage technologies. The surface of the glucose molecule contains a large amount of –OH, making it compatible with many organic substances and be able to grab most of the organic pollutants during the synthesis and convert them to the useful dopants in the porous carbon. Compared with previous methods for making heteroatom‐doped porous carbon, our approach and strategy has the following features: 1) simultaneous treatment and recovery of useful components (such as N and S) from dye wastewater; 2) the elimination of the requirement of additional chemicals to act as N and S sources in order to prepare the heteroatom‐doped carbon; 3) providing a new approach for dye wastewater treatment with a value‐added function; and 4) the resultant carbon material possesses 3D hierarchical structures with a high specific surface area, moderate N/S contents, and a high degree of graphitization, all of which provide a better material for use in energy storage systems.

## 2 Results and Discussion

**Figure 1:**
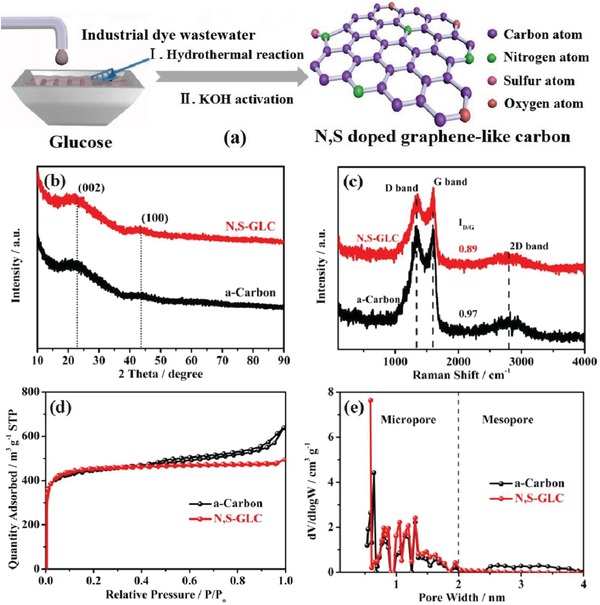
a) Schematic illustration of N,S‐doped graphene‐like carbon. b) XRD patterns of a‐carbon and N,S‐GLC. c) Raman spectra of a‐carbon and N,S‐GLC. d) Nitrogen adsorption–desorption isotherms of a‐carbon and N,S‐GLC. e) Pore size distribution analyzed by the NLDFT method.

The integration of industrial wastewater into the production of N,S‐doped graphene‐like carbon is schematically illustrated in **Figure** **1**a. The dye wastewater used in this study contained mainly azo dye compounds, sulfite (SO_3_^2−^) functional groups, and organic materials that are rich in N and S.^30^, ^31^ The dye wastewater was mixed with glucose prior to the hydrothermal reaction. After the heat treatment, the organic and N/S atoms deposited on the glucose‐based carbon. The dehydration of glucose to oligosaccharides and aromatic compounds occurred mainly at temperatures below 140 °C. Above 160 °C, the oligosaccharide molecules undergo intermolecular dehydration to form a crystal nucleus. These crystal nuclei continue to undergo polymerization and eventually form carbon spheres with active functional groups, such as hydroxyl and carbonyl groups, on the surface. These functional groups not only increase the hydrophilicity of carbon materials, but also adsorb some of the ions in the industrial wastewater to form composite carbon materials. KOH activation was then used to generate more porosity and promote the degree of graphitization.^32^ The reaction between KOH and carbonaceous material can be primarily expressed as

$$6\text{KOH}+2\text{C}\to 2\text{K}+3{\text{H}}_{2}+2{\text{K}}_{2}{\text{CO}}_{3}$$

KOH can transform into potassium carbonate (K_2_CO_3_) when the temperature is around 600 °C. Subsequently, as the temperature increases, the K_2_CO_3_ decomposes to produce potassium oxide (K_2_O) and gaseous carbon dioxide (CO_2_). K_2_O can further react with the char to produce potassium (K), which can be inserted into the carbon structure. After washing with HCl, we obtained the layered structure.^33^ Our novel method of integration can help address the environmental concerns caused by the dye industry, and also provides a new way to prepare N,S‐doped carbon materials for an application in energy storage devices.

Figure 1b shows the X‐ray diffraction (XRD) patterns of a‐carbon and N,S‐GLC. Two peaks at around 2θ = 23° and 43.6° correspond to the lattices of (002) and (100) of graphite, respectively, indicating the presence of graphitic structure in the carbon materials. In Raman spectroscopy (Figure 1c), the D band around 1343 cm^−1^ is disorder‐induced, and the G band at around 1600 cm^−1^ is associated with the C—C bond sp^2^‐hybrized carbon.^34^ The *I*_D_/*I*_G_ ratio is often used to measure the level of disorder in graphite/graphene. The *I*_D_/*I*_G_ ratio for a‐carbon and N,S‐GLC is 0.97 and 0.89, respectively, implying a slightly more disordered structure in the a‐carbon. There also exists a 2D peak at around 2800 cm^−1^ that indicates the existence of layered graphite structures.^35^

Nitrogen sorption isotherms, together with the pore size distributions, are shown in Figure 1c,d. The specific surface area was obtained using the Brunauer–Emmett–Teller (BET) method, and the pore size distribution was analyzed by the nonlocal density functional theory (NLDFT). N,S‐GLC presented as a typical, type I isotherm, indicating that it was a microporous carbon in nature.^36^ Samples of a‐carbon were type IV isotherms, which indicated the presence of both micropores and mesopores in the structure. A pore size distribution plot (Figure 1e) clearly showed that the micropores were disproportionately below 1 nm (≈0.64 nm) in diameter, with some micropores in between 1 and 1.3 nm for both a‐carbon and N,S‐GLC, while the a‐carbon had some mesopores with a broad size range from 2.5 to 4 nm. **Table** **1** summarizes the specific surface area and pore volume of the a‐carbon and N,S‐GLC materials. The specific surface area of a‐carbon (1702 m^2^ g^−1^) and N,S‐GLC (1734 m^2^ g^−1^) was similar, while the pore volume of the a‐carbon (0.89 cm^3^ g^−1^) is slightly larger than that of the N,S‐GLC (0.68 cm^3^ g^−1^) due to the presence of the mesopores.

**Table 1:** Textural characteristics and surface element contents of a‐carbon and N,S‐GLC4

Sample	*S*_BET_ [m^2^ g^−1^]	*V*_total_ [cm^3^ g^−1^]	*D*_pore width_ [nm]	Elemental analysis [at%]			
				C1s	O1s	N1s	S2p
a‐Carbon	1702	0.89	0.58	95.04	4.96	–	–
N,S‐GLC	1734	0.68	0.53	92.93	4.26	2.12	0.69

**Figure 2:**
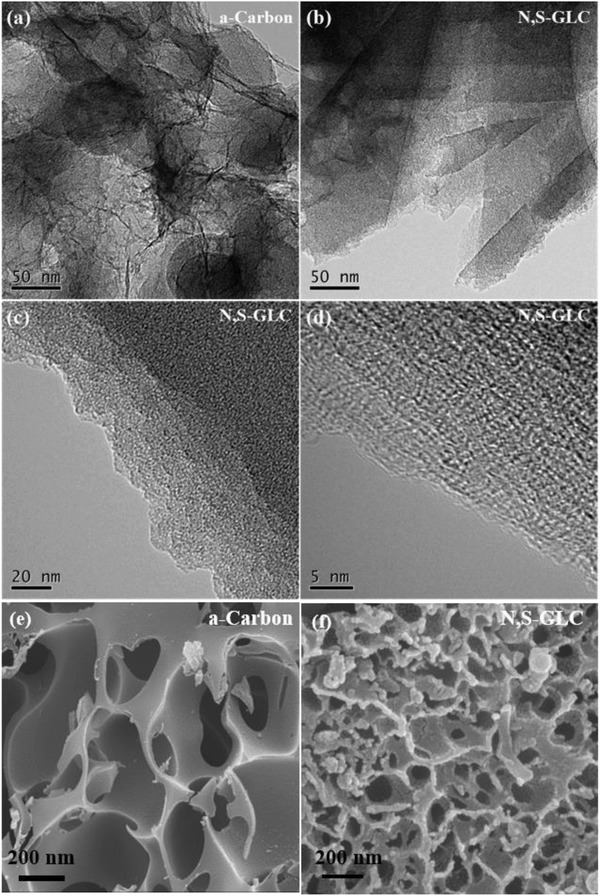
TEM images of a) a‐carbon and b) N,S‐GLC. c,d) High‐resolution TEM image of N,S‐GLC. e) SEM images of a‐carbon and f) N,S‐GLC.

The microstructures and morphologies of the two carbon materials were further characterized using field emission scanning electron microscopy (FESEM) and high‐resolution transmission electron microscopy images (HRTEM) measurements, which are shown in **Figure** **2**a–f. The N,S‐GLC has 3D, interconnected, honeycomb‐like microstructures (Figure 2b,f). The 3D structure with interconnected micropores allows for easy access of the electrolyte to the surface, which shortens the distance of ion transport and minimizes high‐rate diffusional losses.^37^, ^38^ As shown in Figure 2e,f, the pore volume was confirmed by the BET results. A layered structure was seen in the N,S‐GLC, which is consistent with the Raman measurement. Also, segments of graphitic structure could be observed in the HRTEM, indicating that some degree of graphitization exists in the N,S‐GLC despite the dominance of a disordered carbon structure.

**Figure 3:**
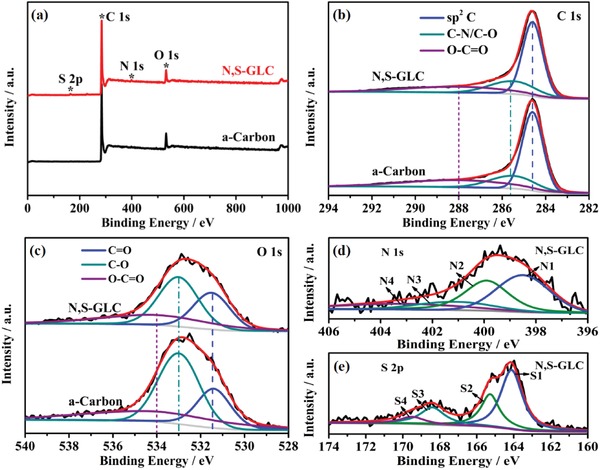
a) XPS survey spectra of a‐carbon and N,S‐GLC. High‐resolution spectra of b) the C1s and c) O1s regions of a‐carbon and N,S‐GLC. High‐resolution spectra of the d) N1s and e) S2p regions of N,S‐GLC.

X‐ray photoelectron spectroscopy (XPS) was used to analyze elements such as C, O, N, and S; additional N and S peaks were observed only in the N,S‐GLC (**Figure** **3**a). The carbon spectrum (Figure 3b) is composed of three peaks. The main peak at 284.6 eV belongs to the sp^2^ graphitic lattice. The peak at 285.5 eV can be assigned to the C—O/C—N functional groups. The peak at 288 eV is attributed to the O—C=O functional groups.^12^, ^38^ The oxygen spectra also consisted of three peaks (Figure 3c). The two main peaks at 531.3 and 533 eV were ascribed to the C=O/S=O and C—O functional groups, respectively. The peak at 534 eV corresponded to the O—C=O functional groups.^39^, ^40^

The nitrogen spectrum of the N,S‐GLC was deconvoluted into four peaks at 398.3, 400, 401, and 403.3 eV, which were assigned to pyridine‐N, pyrrolic‐N, graphitic‐N, and pyridine‐N functional groups, respectively (Figure 3d).^41^, ^42^, ^43^ The sulfur spectrum also contained four peaks (Figure 3e); the peaks at 164.2, 165.28, 168.4, and 169.49 eV can be assigned to the —C—S—C—, —C=S—, and sulfur oxide functional groups, respectively.^20^, ^44^ The atomic ratios based on the XPS analysis are listed in Table 1. The N and S contents of N,S‐GLC are 2.12 and 0.69 at%, respectively, indicating the successful N and S co‐doping of C with N and S sources from the dye wastewater. The high proportion of N and S also confirms the effective treatment of the dye wastewater. The abundant surface N and S functional groups are expected to enhance the electrochemical energy storage capability through improved wettability, a modified density of state (DOS), as well as tuning the Fermi level of the carbon sheet, which would influence the quantum capacitance associated with the EDL capacitance.^45^, ^46^, ^47^ The high specific surface area, good degree of graphitization, 3D interconnected porous structure, and abundant functional groups of the N,S‐GLC are all beneficial for use as a supercapacitor's electrode material.

**Figure 4:**
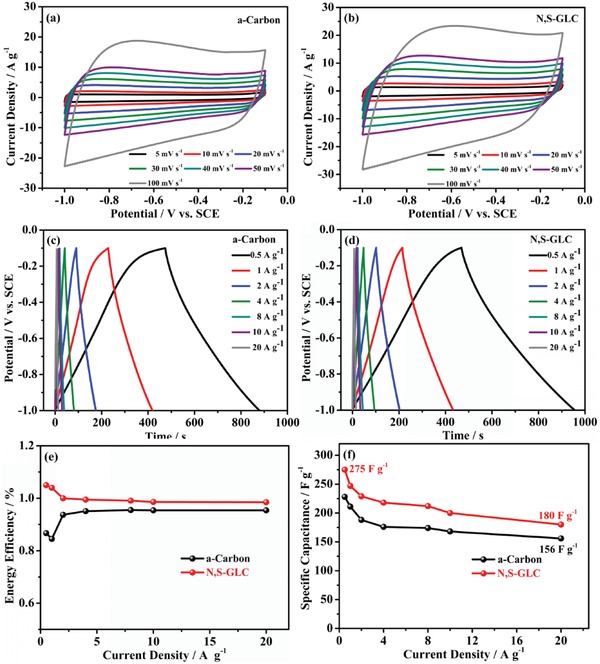
CV curves at different scan rates from 5 to 100 mV s^−1^ for a) a‐carbon and b) N,S‐GLC. GCD curves at current densities in the range of 0.5–20 A g^−1^ for c) a‐carbon and d) N,S‐GLC. e) The columbic efficiency images of prepared a‐carbon and N,S‐GLC. f) Specific capacitances at different current densities from 0.5 to 20 A g^−1^ of a‐carbon and N,S‐GLC measured in a three‐electrode system using a 6 m KOH electrolyte.

The electrochemical performances of a‐carbon and N,S‐GLC were first evaluated in a three‐electrode system with a 6 m KOH as the aqueous electrolyte. cyclic voltammetry (CV) measurements were performed in the potential window from −1 to −0.1 V (vs saturated calomel electrode, SCE). As shown in **Figure** **4**a,b, the shapes of the CV curves were approximately rectangular as the scan rates changed from 5 to 100 mV s^−1^. The patterns do not show significant polarization and changes at high sweep speeds, indicating a good and stable ability to charge–discharge. The current density of the N,S‐GLC was higher than that of a‐carbon at the same scan rate, implying a higher specific capacitance of the N,S‐GLC.

Figure 4c,d displays the charge–discharge curves of a‐carbon and N,S‐GLC at current densities ranging from 1 to 20 A g^−1^. The charge–discharge curves for the two electrodes were similar. The shape of the charge–discharge curves remained quasi‐triangular and symmetrical even at a high current density of 20 A g^−1^, indicating that the electrode material exhibits good capacitive behavior. The galvanostatic charge–discharge (GCD) results are also in line with the CV tests. The columbic efficiency was also calculated based on the ratio of discharge/charge times. As shown in Figure 4e, the N,S‐GLC displayed a higher efficiency than the a‐carbon.

Figure 4f summarizes the specific capacitance values of a‐carbon and N,S‐GLC that were calculated from the charge–discharge curves. N/S co‐doped N,S‐GLC had a good capacitance retention. The maximum specific capacitance of N,S‐GLC is 275 F g^−1^ at a current density of 0.5 A g^−1^, which is superior to recent reports of N and S co‐doped carbon materials (**Table** **2**). Moreover, at a higher current density of 20 A g^−1^, the capacitance was still as high as 180 F g^−1^, suggesting a capacitance retention up to 65.4%. Although the N,S‐GLC had a moderate specific surface area, the surface N and S functional groups helped enhance the wettability, and the good degree of graphitization improved the conductivity of the electrode material.

**Table 2:** Comparisons of different heteroatom‐doped carbon for supercapacitor electrode materials

Sample	N/S source [s]	*C*_m_ [F g^−1^]	Current density [A g^−1^]	Electrolyte	Energy density	Power density	Ref.
PCNs1‐1	Thiourea	249	0.5	6 m KOH	21 Wh kg^−1^	180 W kg^−1^	20
ADGC‐1:1	Thiourea	127	10	1 m H_2_SO_4_	9.04 Wh kg^−1^	288 W kg^−1^	48
NSOMC	Pyrrole, H_2_SO_4_	186	0.2	6 m KOH	N/A	N/A	19
NS‐PCMSs	Thiourea	247	10	6 m KOH	N/A	N/A	49
NS‐HGH	NH_4_SCN	209	10	6 m KOH	24.7 Wh kg^−1^	N/A	21
NSPCS	N,S polymer	230	1	1 m H_2_SO_4_	N/A	N/A	18
N‐ICNs	Dandelion seeds	337	1	6 m KOH	25.3 Wh kg^−1^	900 W kg^−1^	50
B/N‐PCTBs	Urea, boric	335	1	6 m KOH	12.15 Wh L^−1^	699.84 W L^−1^	51
N,S‐GLC	Industrial dye wastewater	275	0.5	6 m KOH	6.4 Wh kg^−1^	275 W kg^−1^	This study

**Figure 5:**
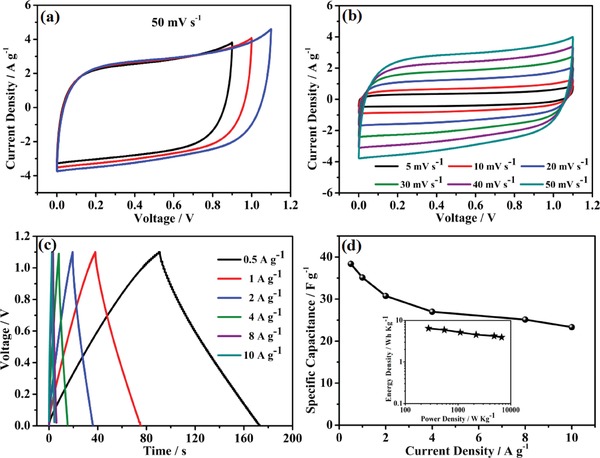
Electrochemical performance of a N,S‐GLC//N,S‐GLC symmetric supercapacitor. a) The CV curves of the symmetrical supercapacitor in different operation voltages at a scan rate of 50 mV s^−1^. b) The CV curves during operation voltages of 1.1 V at different scan rates. c) The galvanostatic charge–discharge curves at different current densities. d) Specific capacitances calculated from the GCD curves and a Ragone plot of the symmetric supercapacitor (insert).

The electrochemical performance of the N,S‐GLC sample was further tested in a two‐electrode system. As shown in **Figure** **5**a, we first adjusted the voltage window of the electrochemical system and then gradually expanded the voltage window from 0.9 to 1.1 V. We saw that the shapes of the CV curve did not change significantly, and it remained a rectangular shape. No significant polarization occurs at one end of the voltage, indicating that the cell can perform over a voltage window of 1.1 V. Figure 5b shows the cyclic voltammogram curves at sweep speeds ranging from 5 to 50 mV s^−1^ over the 1.1 V voltage window. The shape of the curves remained constant as the sweep rate increased, indicating good capacitive processes.

The galvanostatic charge–discharge curves of the symmetric supercapacitor are shown in Figure 5c. The triangular shape of the charge–discharge curves remained even as the current densities increased from 0.5 to 10 A g^−1^. There was also no obvious voltage drop at a higher current density, indicating a small equivalent series resistance. Since the electrolyte used was KOH, pseudo contribution was not obvious, but the presence of the heteroatoms (N and S) did improve the wettability of the surface, tuned the Fermi‐level of the graphene, and improved the DOS of the thin carbon sheet (graphene sheets), which significantly improves the quantum capacitance of the electrode.^44^, ^48^, ^49^ The capacitance of the symmetric supercapacitor system is shown in Figure 5d. A capacitance of 38 F g^−1^ was obtained at a current density of 0.5 A g^−1^. When the current density was increased to 10 A g^−1^, the capacitance of the system remained at about 23 F g^−1^, corresponding to a power density of 6.7 kW kg^−1^, and a capacitance retention rate of 60%. The satisfactory electrochemical capacitive performance showed that the synthesis of N,S co‐doped porous carbon via the recovery of N and S from dye wastewater is a promising green and sustainable approach.

## 3 Conclusions

In summary, we successfully prepared an N and S co‐doped graphene‐like porous carbon via an efficient hydrothermal reaction of glucose with dye wastewater followed by KOH activation. The prepared N,S‐GLC possessed interconnected porosity, moderate specific surface area (1734 m^2^ g^−1^), rich N and S contents (2.12 and 0.69 at%, respectively), and a good degree of graphitization. When used as an electrode material for a supercapacitor, the N,S‐GLC displayed a high specific capacitance of 275 F g^−1^ at a current density of 0.5 A g^−1^ and a good rate capability with a retention of 65.4% at 20 Ag^−1^ in 6 m KOH. Moreover, the assembled symmetrical supercapacitor device showed a capacitance of 38 F g^−1^ at a current density of 0.5 A g^−1^, which is equivalent to an energy density of 6.4 Wh kg^−1^ with a power density of 275.0 W kg^−1^. These electrochemical results show that N,S‐GLC is a promising candidate for energy storage applications. The synthesis strategy developed in this work could also provide a route to recover energy from dye wastewater.

## 4 Experimental Section

*Preparation of N,S‐GLC*: To prepare the N,S‐GLC, glucose (2 g) was first added into 50 mL of industrial dye wastewater (Xinjiang Shenbang Environmental Engineering Co., Ltd.) and then the solution was mixed using ultrasonication for 10 min. Secondly, the mixture was moved to Teflon‐lined autoclave for the hydrothermal reaction and kept at 180 °C for 10 h. Finally, the black precursor was collected and thoroughly washed with deionized water before drying at 80 °C for 8 h. 1 g of the dried, black precursor was mixed with 1 g KOH in an agent mortar for complete grinding. The ground mixture was then activated at a rate of 5 °C min^−1^ and kept at 700 °C for 1 h in an argon atmosphere. The activated carbon was first washed with 10% (v/v) HCl followed by abundant deionized water to get to a pH of 7.0, and the final doped carbon (N,S‐GLC) was dried at 80 °C for 8 h.

As a control, the same experiment conditions were used but deionized water was used in lieu of the dye wastewater and a dark brown precursor was obtained. The dark brown precursor was activated with KOH and the final product was named a‐carbon.

*Physical Characterization*: XRD was performed with a Bruker D8 Advanced X‐ray diffractometer furnished with a Cu Kα radiation filter (λ = 1.5147 Å). The FESEM surveys were examined using a Hitachi SU8010 instrument. A field emission FEI Tecnai G2 F20 TEM was used to investigate the morphology of the carbon materials. The nitrogen adsorption and desorption was tested with a Micromeritics Instrument 3Flex comptometer. The specific surface area was analyzed by the BET method, and the pore size distribution was analyzed with a *t*‐plot method and NLDFT. The XPS spectra was characterized by an ESCALAB 250Xi (Thermo Fisher Scientific) spectrometer using monochromatic Al Kα radiation. Raman spectra were collected using a Horiba Jobin Yvon LabRAM HR800 Raman spectrometer.

*Electrochemical Characterization*: The a‐carbon and N,S‐GLC materials were first tested using a three‐electrode system. A platinum plate was used as the counter electrode and an SCE was used as the reference electrode. The electrolyte was a 6 m KOH solution. The working electrode was prepared as follows: the as‐prepared carbon material (5 mg) was mixed with polytetrafluoroethylene (PTFE,1 µL) and acetylene black (1 mg), and then 1 mL of ethanol was added into the mixture. The mixture was agitated via ultrasonication for at least 40 min to get an inky liquid. The inky liquid was then coated evenly onto a nickel foam (1 cm × 1 cm) and dried at 80 °C for 10 h before being pressed at 20 MPa for 1 min to obtain the working electrodes. The electrochemical tests were conducted on a CHI 760E workstation at 25 °C using CV and GCD measurements. CV curves were tested in the −0.9 to 0 V range at a variety of scan rates ranging from 5 to 50 mV s^−1^. GCD tests were measured in the potential range of −0.9 to 0 V at current densities from 0.5 to 20 A g^−1^. The specific capacitance was calculated using the discharge curves with the following equation

(1) C=IΔt/mΔV

where *C* is the specific capacitance (F g^−1^), *I* is the current density (A), *m* is the mass in g of the electrode material, Δ*t* is the discharge time (s), and Δ*V* is the operating potential in *V* of the three‐electrode system.

The electrode material with the best performance was further tested using a symmetrical two‐electrode system. The inky liquid mixture was evenly coated on a circular nickel foam (0.785 cm^2^). It was obtained by vacuum drying at 80 °C for 10 h and then pressed at 20 MPa for 1 min to obtain the working electrodes. The two electrodes were assembled by using a CR2032 stainless steel coin cell with a cellulose membrane and a 6 m KOH electrolyte. The electrochemical performance was tested using the CV/GCD method. The specific capacitance was calculated using Equation (1). The energy densities and powder densities were calculated using the following equations

(2) $$E=C{\left(\Delta V\right)}^{2}\text{/}\left(8\times 3.6\right)$$

(3) $$P=\left(E\times 3600\right)\text{/}t$$

where *E* is the specific energy density (Wh kg^−1^), *P* is the specific power density of the symmetrical supercapacitor system (W kg^−1^), *C* is the specific capacitance of the total symmetrical system (F g^−1^), and Δ*V* is the cell voltage for charging and discharging.

## Conflict of Interest

The authors declare no conflict of interest.

## References

[1] L. L. Zhang , X. S. Zhao , Chem. Soc. Rev. 2009, 38, 2520. 19690733 10.1039/b813846j

[2] T. Qin , Z. Wan , Z. Wang , Y. Wen , M. Liu , S. Peng , D. He , J. Hou , F. Huang , G. Cao , J. Power Sources 2016, 336, 455.

[3] Z. Gao , C. Bumgardner , N. Song , Y. Zhang , J. Li , X. Li , Nat. Commun. 2016, 7, 11586. 27189776 10.1038/ncomms11586 PMC4873971

[4] X. Wu , L. Jiang , C. Long , Z. Fan , Nano Energy 2015, 13, 527.

[5] C. Long , D. Qi , T. Wei , J. Yan , L. Jiang , Z. Fan , Adv. Funct. Mater. 2014, 24, 3953.

[6] G. Zhao , C. Chen , D. Yu , L. Sun , C. Yang , H. Zhang , Y. Sun , F. Besenbacher , M. Yu , Nano Energy 2018, 47, 547.

[7] X. Zang , R. Zhang , Z. Zhen , W. Lai , C. Yang , F. Kang , H. Zhu , Nano Energy 2017, 40, 224.

[8] X. Li , B. Wei , Nano Energy 2013, 2, 159.

[9] L. Zhang , D. Huang , N. Hu , C. Yang , M. Li , H. Wei , Z. Yang , Y. Su , Y. Zhang , J. Power Sources 2017, 342, 1.

[10] Z. Peng , J. Lin , R. Ye , E. L. Samuel , J. M. Tour , ACS Appl. Mater. Interfaces 2015, 7, 3414. 25584857 10.1021/am509065d

[11] Y. Hu , Y. Zhao , Y. Li , H. Li , H. Shao , L. Qu , Electrochim. Acta 2004, 49, 279.

[12] B. Liu , L. Zhang , P. Qi , M. Zhu , G. Wang , Y. Ma , X. Guo , H. Chen , B. Zhang , Z. Zhao , Nanomaterials 2016, 6, 18. 10.3390/nano6010018 PMC5302551 28344275

[13] C. Tran , D. Lawrence , F. W. Richey , C. Dillard , Y. A. Elabd , V. Kalra , Chem. Commun. 2015, 51, 13760. 10.1039/c5cc04359j 26234368

[14] C. Wang , Y. Zhou , L. Sun , P. Wan , X. Zhang , J. Qiu , J. Power Sources 2013, 239, 81.

[15] Y. Zhang , X. Liu , S. Wang , L. Li , S. Dou , Adv. Energy Mater. 2017, 7, 1700592.

[16] M. Salanne , B. Rotenberg , K. Naoi , K. Kaneko , P. L. Taberna , C. P. Grey , B. Dunn , P. Simon , Nat. Energy 2016, 1, 16070.

[17] A. C. Forse , C. Merlet , J. M. Griffin , C. P. Grey , J. Am. Chem. Soc. 2016, 138, 5731. 27031622 10.1021/jacs.6b02115 PMC4865825

[18] R. S. Mehare , S. P. Ranganath , V. Chaturvedi , M. V. Badiger , M. V. Shelke , Energy Fuels 2017, 32, 908.

[19] D. Zhang , M. Han , B. Wang , Y. Li , L. Lei , K. Wang , Y. Wang , L. Zhang , H. Feng , J. Power Sources 2017, 358, 112.

[20] Y. Li , G. Wang , T. Wei , Z. Fan , P. Yan , Nano Energy 2016, 19, 165.

[21] W. Kong , J. Zhu , M. Zhang , Y. Liu , J. Hu , Microporous Mesoporous Mater. 2018, 268, 260.

[22] C. Wang , X. Wang , H. Lu , H. Li , X. S. Zhao , Carbon 2018, 140, 139‐147.

[23] H. Lu , X. S. Zhao , Sustainable Energy Fuels 2017, 1, 1265.

[24] H. Lu , X. Sun , R. R. Gaddam , N. A. Kumar , X. S. Zhao , J. Power Sources 2017, 360, 634.

[25] E. Viau , K. Bibby , T. Paezrubio , J. Peccia , Environ. Sci. Technol. 2011, 45, 5459. 21644497 10.1021/es200566f

[26] A. Kelessidis , A. S. Stasinakis , Waste Manage. 2012, 32, 1186. 10.1016/j.wasman.2012.01.012 22336390

[27] M. M. Khin , Energy Environ. Sci. 2012, 5, 8075.

[28] E. M. Iannicelli‐Zubiani , P. Gallo Stampino , C. Cristiani , G. Dotelli , Chem. Eng. J. 2018, 341, 75.

[29] A. F. Hassan , H. Elhadidy , J. Environ. Chem. Eng. 2017, 5, 955.

[30] L. Fan , N. Zhang , K. Sun , RSC Adv. 2014, 4, 21419.

[31] S. J. Yuan , X. H. Dai , Sci. Rep. 2016, 6, 27570. 27273314 10.1038/srep27570 PMC4895130

[32] W. Qian , F. Sun , Y. Xu , L. Qiu , C. Liu , S. Wang , F. Yan , Energy Environ. Sci. 2013, 7, 379.

[33] J. Wang , S. Kaskel , J. Mater. Chem. 2012, 22, 23710.

[34] Q. Wang , J. Yan , Y. Wang , T. Wei , M. Zhang , X. Jing , Z. Fan , Carbon 2014, 67, 119.

[35] X. Liu , D. Chao , Y. Li , J. Hao , X. Liu , J. Zhao , J. Lin , H. J. Fan , Z. X. Shen , Nano Energy 2015, 17, 43.

[36] H. Y. Liu , K. P. Wang , H. Teng , Carbon 2005, 43, 559.

[37] L. Xie , G. Sun , F. Su , X. Guo , Q. Q. Kong , X. M. Li , X. Huang , L. Wan , W. Song , K. Li , J. Mater. Chem. A 2015, 4, 1637.

[38] N. D. Kim , D. B. Buchholz , G. Casillas , M. José‐Yacaman , R. P. H. Chang , Adv. Funct. Mater. 2014, 24, 4186.

[39] Y. Huang , L. Peng , Y. Liu , G. Zhao , J. Y. Chen , G. Yu , ACS Appl. Mater. Interfaces 2016, 8, 15205. 27220422 10.1021/acsami.6b02214

[40] Y. Wang , M. Zhu , G. Wang , B. Dai , F. Yu , Z. Tian , X. Guo , Nanomaterials 2017, 7, 404. 10.3390/nano7110404 PMC5707621 29165362

[41] L. Shi , T. Wu , Y. Wang , J. Zhang , G. Wang , J. Zhang , B. Dai , F. Yu , Materials 2017, 10, 1030. 10.3390/ma10091030 PMC5615685 28869543

[42] Y. Wang , M. Zhu , Y. Li , M. Zhang , X. Xue , Y. Shi , B. Dai , X. Guo , F. Yu , Green Energy Environ. 2018, 3, 172.

[43] Y. Wang , F. Yu , M. Zhu , C. Ma , D. Zhao , C. Wang , A. Zhou , B. Dai , J. Ji , X. Guo , J. Mater. Chem. A 2018, 6, 2011.

[44] Y. Huang , S. L. Candelaria , Y. Li , Z. Li , J. Tian , L. Zhang , G. Cao , J. Power Sources 2014, 252, 90.

[45] J. Xia , F. Chen , J. Li , N. Tao , Nat. Nanotechnol. 2009, 4, 505. 19662012 10.1038/nnano.2009.177

[46] H. W. Liang , X. Zhuang , Brüller, Sebastian , X. Feng , K. Müllen , Nat. Commun. 2014, 5, 4973. 25229121 10.1038/ncomms5973

[47] R. Furue , T. Nishimoto , I. S. Park , J. Lee , T. Yasuda , Angew. Chem., Int. Ed. 2016, 55, 7171. 10.1002/anie.201603232 27145481

[48] C. Chang , X. Yang , S. Xiang , X. Lin , H. Que , M. Li , J. Electrochem. Soc. 2017, 164, A1601.

[49] J. Zhang , J. Zhou , D. Wang , L. Hou , F. Gao , Electrochim. Acta 2016, 191, 933.

[50] J. Zhao , J. Gong , Y. Li , K. Cheng , K. Ye , K. Zhu , J. Yan , D. Cao , G. Wang , Acta Chim. Sin. 2018, 76, 107.

[51] J. Zhao , Y. Li , G. Wang , T. Wei , Z. Liu , K. Cheng , K. Ye , K. Zhu , D. Cao , Z. Fan , J. Mater. Chem. A 2017, 5, 23085.

